# Plant functional diversity promotes ecosystem multifunctionality in abandoned karst farmland under changing rainfall patterns

**DOI:** 10.3389/fpls.2026.1883399

**Published:** 2026-07-13

**Authors:** Yuancai Qi, Maji Wan, Jinchun Liu, Shehzadi Kiran, Weixue Luo, Jianping Tao

**Affiliations:** 1 Key Laboratory of Eco-environments in the Three Gorges Reservoir Region, Ministry of Education, School of Life Sciences, Southwest University, Chongqing, China; 2 Chongqing Jinfo Mountain Karst Ecosystem National Observation and Research Station, Southwest University, Chongqing, China

**Keywords:** abiotic factors, functional diversity, karst farmland, multifunctionality, rainfall frequency

## Abstract

**Introduction:**

Global climate change is altering the frequency and intensity of rainfall, with consequent effects on ecosystem multifunctionality (EMF). However, under more frequent extreme rainfall conditions, it remains unclear whether abiotic and biotic factors exert identical effects on EMF, and what the primary drivers of EMF are.

**Methods:**

In this study, we quantified EMF using both averaging and multiple-threshold approaches. Within the framework of Hill-Chao numbers, plant biodiversity and soil microbial diversity were measured. We explored the effects of abiotic factors, plant diversity, and soil microbial diversity on EMF in abandoned karst farmland by simulating changes in the frequency of rainfall events of varying intensities. By manipulating rainfall frequency across different intensities while maintaining natural annual rainfall totals, we assessed its influence on multifunctionality and identified the primary drivers of variation.

**Results:**

Increased frequency of heavy rainfall reduced plant biodiversity but had positive effects on the maintenance of EMF, although these effect were not significant (*P* > 0.05). Among abiotic factors, soil moisture content exhibited a positive relationship with EMF. Notably, plant functional diversity emerged as a primary driver, with its positive effect on EMF strengthening at higher functional thresholds (e.g., EMF_T50_ and EMF_T90_). Furthermore, abiotic factors affected EMF through both direct and indirect pathways, with the indirect effects primarily mediated by functional diversity, particularly under high-threshold multifunctionality.

**Discussion:**

These findings suggest that, under projected future increases in extreme rainfall events, enhancing plant functional diversity should be a priority during the early succession of abandoned karst farmland. Strategies aimed at increasing plant functional diversity may benefit the restoration of ecosystem functions in degraded landscapes.

## 1 Introduction

Precipitation is one of the most critical factors in shaping and maintaining the structure and function of ecosystems, with even slight changes in rainfall causing dramatic changes in ecosystem functions (Nielsen and Ball, 2015; Shi et al., 2023). A large number of evidence from model projections, controlled experiments, and empirical observations indicate an increasing frequency of extreme precipitation events across many regions, a trend projected to persist longtime in the future (Boers et al., 2019; Wang et al., 2020). These extreme rainfall events are characterized by heightened rainfall intensity, reduced rainfall frequency, and extended drought intervals, despite relatively stable annual precipitation totals (Gherardi and Sala, 2015; Ishii et al., 2017; Zhang and Zhao, 2022). Despite extensive research on ecosystem responses to changes in total annual rainfall, how ecosystems respond to an altering frequency and intensity of rainfall (hereafter referred to as “altered rainfall patterns”) under conditions of constant annual precipitation remains largely unexplored (Heisler-White et al., 2009).

Altered rainfall patterns can influence key ecosystem functions, such as primary productivity, carbon sequestration, and nutrient cycling, by modifying soil water availability (Zhou et al., 2022). For instance, extreme rainfall events often prolong drought intervals, reducing soil water availability, which in turn weakens both ecosystem stability and biodiversity, ultimately altering functions like biomass production (Li et al., 2018). Conversely, extreme precipitation may also trigger divergent responses in plant functional traits (e.g., specific leaf area, root depth), leading to shifts in functional diversity, while drive the emergence of novel ecosystem functional regimes under extreme precipitation scenarios (Cheng et al., 2021; Zhang et al., 2023). These trait and diversity mediated changes in species growth, reproduction, and resource use provide a context for testing competing ecological hypotheses. Specifically, the niche complementarity hypothesis, which posits that greater biodiversity enhances ecosystem functions through resource partitioning and facilitation, tends to receive more support than the mass ratio hypothesis, which emphasizes the dominant species traits, under conditions of extreme rainfall (Gao et al., 2021; Kelemen et al., 2017). Collectively, the above findings suggest a potential trade-off in how different facets of biodiversity, like species richness and functional diversity, drive ecosystem functions, a trade-off that may intensify with prolonged periods of extreme rainfall and drought (Gherardi and Sala, 2015; Oliver et al., 2015). Therefore, further experimental studies are needed to elucidate the processes and mechanisms of this trade-off, with particular emphasis on the relative contributions of biodiversity to the maintenance of ecosystem functions.

Furthermore, abiotic factors can directly regulate ecosystem functions, independently of or in conjunction with biotic factors. Changes in abiotic factors can simultaneously influence the rates and trajectories of multiple ecosystem processes (Manzoni et al., 2014; Delgado-Baquerizo et al., 2020). However, the relative importance of abiotic and biotic drivers may vary depending on the environmental context and disturbance regimes (Jing et al., 2015; Le Bagousse-Pinguet et al., 2019). In water-limited systems, specific abiotic factors, namely rainfall frequency, soil water content (SWC), and soil pH, are often assumed to be the primary direct drivers of ecosystem processes (Feldman et al., 2024; Zhang et al., 2026). It is important to clarify that these factors are external climatic or soil chemical properties, distinct from the internal soil variables that are typically used to drive ecosystem functions. Nevertheless, how these abiotic factors sustain or constrain multiple functions simultaneously under changing rainfall regimes remains a critical knowledge gap. Elucidating the direct role of abiotic factors is essential for unraveling the complex mechanisms underlying ecosystem responses to climate extremes.

Ecosystem functions include a suite of key indicators, such as productivity, soil nutrient availability, nutrient cycling, and carbon sequestration, etc (Edlinger et al., 2020; Weiskopf et al., 2022). However, trade-offs or synergies among these functions are common (Soliveres et al., 2016), implying studies focusing solely on single ecosystem function may fail to fully capture the full impact of changing rainfall patterns, potentially leading to incomplete or misleading conclusions (Edlinger et al., 2020). Ecosystem multifunctionality (EMF), which quantifies the ability of an ecosystem to provide and maintain multiple functions and services simultaneously (Hector and Bagchi, 2007; Manning et al., 2018), might be a more comprehensive indicator for measuring overall ecosystem functions. In karst regions, changing rainfall patterns alter the periodic soil water availability and prolong drought intervals, thereby significantly affecting plant and microbial diversity, which in turn regulates a range of ecosystem functions (Fay et al., 2008; Fry et al., 2014). Despite its importance, it remains unclear whether rainfall-induced changes in soil hydrology and biodiversity will increase or decrease single function, ultimately increasing or decreasing EMF in these vulnerable ecosystems.

Karst landforms, covering approximately 12%-15% of the Earth’s terrestrial surface, constitute a globally significant ecosystem that provides vital ecological functions (Wang et al., 2019; Ding et al., 2021). These ecosystems are particularly vulnerable to environmental changes due to inherent characteristics, including thin soil layers, high permeability, and low water retention capacity (Gutiérrez et al., 2014). A large body of literatures has shown that karst ecosystems are especially sensitive to variations in the timing, duration, and amount of precipitation (Gao et al., 2020; Zhao et al., 2020; Liu et al., 2023; Suolang et al., 2024). Increased precipitation intensity, even if the annual total amount remains unchanged, can prolong inter-rainfall event intervals, inducing soil surface drought conditions that may suppress plant growth and even cause mortality, particularly among shallow-rooted herbaceous species in karst areas (Miguez-Macho and Fan, 2021; Huang et al., 2022). However, under extreme rainfall conditions, karst systems exhibit contrasting microbial responses, where dominant soil microbial taxa shift, and the abundance of functional genes increase, accelerating the turnover of certain soil elements compared to natural rainfall conditions (Suolang et al., 2024). Given that rainfall changes may exert contrasting effects on karst ecosystem functions, further experimental studies are urgently needed to clarify how extreme rainfall shapes functions.

According, we established a partial rainout shelter in an abandoned karst farmland, and conducted a series of rainfall manipulation treatments. This study addressed two scientific questions: (1) How do changing rainfall patterns affect aboveground and underground biodiversity? and (2) Under changing rainfall patterns, which biotic and abiotic factors have a greater impact on EMF? Based on the current state of research, we hypothesize: (1) rainstorm frequency enhanced treatment would initially restrain plan diversity and soil microbial diversity in the initial stage of abandoned karst farmland, and (2) abiotic factors can directly regulate the EMF, and may also indirectly drive the EMF through functional diversity.

## 2 Materials and methods

### 2.1 Study area

The research area is located at Zhongliang Mountain, which serves as an auxiliary observation field for the Chongqing Jinfo Mountain Karst Ecosystem National Observation and Research Station, China (106° 26′ 47.72” E, 29° 47′ 4.75” N), with an altitude of 592 m. The region experiences a subtropical monsoon humid climate, characterized by hot, humid summers and foggy winters. The average temperature ranges from 16.8 to 18.0 °C, and the average annual precipitation is about 1000–1300 mm, with the majority falling between May to September (Chen et al., 2021)(**Supplementary Figure 1**). The annual potential evapotranspiration is approximately 878 mm (Xiong et al., 2013). As a typical karst mountainous region, the dominant vegetation is subtropical evergreen broad-leaved forests and shrublands. The soil layer is thin and uneven, with numerous rock fissures. Factors such as lithology, soil characteristics, soil erosion, and human activity have contributed to the absence of a typical evergreen broad-leaved forest in this karst region. Prior to the experiment, the soil pH was 5.30, with total nitrogen of 1.54 g/kg, total carbon of 12.93 g/kg, organic matter of 25.11 g/kg, and alkali-hydrolyzable nitrogen of 139.92 mg/kg. For the purpose of this study, a relatively flat experimental site was constructed on an abandoned karst farmland. Before abandonment, the farmland was primarily cultivated with maize and wheat. In the years of abandonment, no crops were planted in the plots, and the ground vegetation consisted of naturally regenerated herbaceous plant communities.

### 2.2 Precipitation control

**Figure 1:**
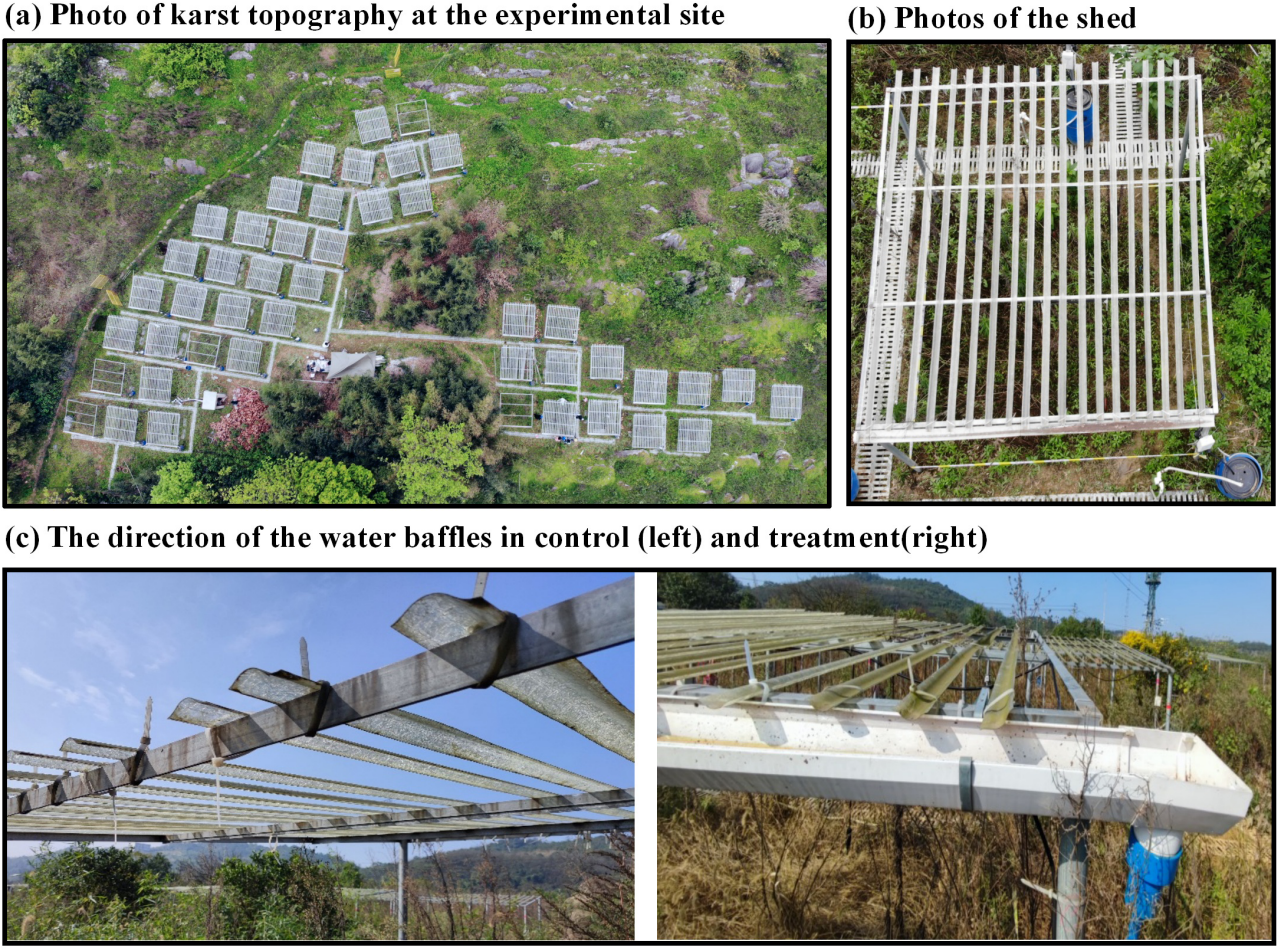
**(a)** Photo of karst topography at the experimental site. **(b)** Photos of individual sheds. **(c)** The placement direction of the water baffles in the control group (left) and the rainfall treatment group (right).

The precipitation control platform was established in 2019, including a rainfall shelter system, a rainfall interception and collection system, and an intercepted rainwater return system. In total, 40 experimental sheds (3m×3m each) were built (**Figure 1a**). To prevent soil water exchange between different adjacent plots, impermeable barriers were installed around them. Prior to the experiment, we dug out the 20 cm depth of topsoil from the experimental area, removed the plants and roots from the soil, then uniformly mixed and backfilled the soil in each plot. For rainfall manipulation system, 19 V-shaped baffles were installed above each treatment shelter with their grooves facing upward to intercept 40% of rainwater (**Figure 1b**). The intercepted rainwater was stored in a tank and later returned to the same shelter according to special thresholds, forming four precipitation treatments: (1) Moderate rainfall frequency enhanced treatment (MR): when the intercepted rainfall reaches 10 mm (equivalent to a water volume of 0.09 m^3^ in the tank), immediately replenish it with moderate rain; (2) Heavy rainfall frequency enhanced treatment (HR): when the intercepted rainfall reaches 25 mm (equivalent to a water volume of 0.225 m^3^ in the tank), immediately replenish it with heavy rain; and (3) Rainstorm frequency enhanced treatment (SR): when the intercepted rainfall reaches 50 mm (equivalent to a water volume of 0.45 m^3^ in the tank), immediately replenish it with rainstorm; (4) Natural rainfall as the control treatment (CK), 19 V-shaped baffles were installed above each shelter with their grooves facing downward in natural rainfall shelters and did not intercept rainwater (**Figure 1c**). All experimental water storage tanks were equipped with water level sensors to timely monitor stored rainfall volume. Once a predefined replenishment threshold was reached, a 12V pump automatically activated to replenish rainwater. The system maintained a constant total annual precipitation while manipulating the intensity and frequency of individual rainfall events. The precipitation treatments were initiated in September 2020, with 10 replicates per treatment. Further operational details can be found in Suolang (2024). This experimental design successfully made significant differences in rainfall frequency and the single rainfall amount (**Supplementary Figure 1**).

### 2.3 Sample collection and determination

In August 2021, each 3m×3m plot was divided into four 1.5m×1.5m quadrats. Within each quadrat, all plant species were identified, and their heights, coverage, and abundance were observed. The results of the vegetation survey under different rainfall treatment was shown in **Supplementary Table 1**. The aboveground and underground biomass was estimated using the harvested method. Soil samples from the 0–20 cm depth were collected according to a five-point sampling method. Prior to the formal experiment, we homogenized the soil composition across all plots through thorough mixing. For each rainfall treatment, soil samples were collected from four randomly selected replicate plots (n = 4 per treatment). Within each plot, five soil cores were taken using a five-point sampling method (four corners and the center) at a depth of 0–20 cm, and then thoroughly mixed to form one composite sample per plot. Each composite sample was then divided into three subsamples: one subsample was air-dried for analysis of soil physicochemical properties; the second subsample was stored at -20 °C for subsequent determination of soil extracellular enzyme activities; and the third subsample was stored at -80 °C for soil microbial community identification. Since the soil was uniformly mixed before the experiment, the baseline soil values were considered identical across all treatments. Variations in soil properties among treatments were primarily attributed to different rainfall conditions. Therefore, the soil data for the remaining six plots in each treatment were replaced by the average values of the four plots within that treatment.

We measured EMF based on four types of ecosystem functions (e.g. plant productivity, carbon cycling, nitrogen cycling, and phosphorus cycling). Specifically, plant productivity (PP) indicators included aboveground biomass (AGB) and underground biomass (BGB). Carbon cycle (CC) indicators included soil organic carbon (SOC), microbial biomass carbon (MBC), and sucrase (S-SC). Nitrogen cycle (NC) indicators included soil total nitrogen (TN), soil microbial biomass nitrogen (MBN), nitrate nitrogen (NO_3_^--^N), ammonium nitrogen (NH_4_^+^-N), inorganic nitrogen (IN), and alkali-hydrolyzed nitrogen (AN). Phosphorus cycle (PC) indicators included soil total phosphorus (TP), microbial biomass phosphorus (MBP), residual phosphorus (RP), acid phosphatase (ACP), and phosphodiesterases (PDEs). All measured indices were closely related to plant productivity, the carbon cycle, the nitrogen cycle and the phosphorus cycle (**Supplementary Table 2**). ABM and BGM were measured by oven-drying plant materials at 80°C until constant weight. Ra, Rh, and Rs were determined using the static chamber method and the root division method (Yue et al., 2018). SOC was quantified through potassium dichromate oxidation (Song et al., 2022). MBC, MBN, and MBP were determined via chloroform fumigation followed by potassium sulfate extraction (Oberson et al., 1997; Song et al., 2022). S-SC was determined by 3,5-dinitrosalicylic acid colorimetric analysis (Yang et al., 2020). TN was measured using an elemental analyzer (Vario EL cube, Elementar, Langenselbold, Germany). TP was measured using an inductively coupled plasma emission spectrometer (ICP-OES, ThermoFisher Icap 6300, UK) (Olsen and Sommers, 1982). Both NH_4_^+^-N and AN were determined by the Alkali Hydrolysis-Diffusion method, while NO_3_^−^-N was determined by UV spectrophotometry. RP was measured using a colorimetric method (Olsen and Sommers, 1982). Acid phosphatase (ACP) activity was measured using the Tabatabai method (Tabatabai, 1994). Detailed measurement methods for these indicators were provided in the Supporting material.

Abiotic factors included rainfall frequency (RF), soil water content (SWC), and soil pH. SWC was determined by oven-drying 10 g of fresh soil at 105 °C until constant weight. Soil pH was determined in a 1:5 mixture of air-dried soil and deionized water using a pH meter (PhS - 3E, Lei Magnetic, Shanghai, China). Furthermore, functional traits included specific leaf area, leaf thickness, root length, root dry matter content, stem dry matter content, and stem tissue density. Leaf area (one-side) and leaf fresh mass (after full rehydration) were measured prior to oven-drying leaves at 65 °C for 48h. Specific leaf area was calculated as the ratio of fresh leaf area to dry mass. Leaf thickness was measured with a leaf thickness gauge. Root length was determined using a root scanner, after which the roots were oven-dried to constant weight to obtain root dry weight. Specific root length was calculated as the ratio of root length to root dry weight. Healthy and mature fresh plant stems were weighed using an electronic balance (precision 0.0001 g) and then oven-dried to constant weight to obtain stem dry weight. The stem dry matter content was derived from the ratio of stem dry weight to fresh weight. Stem tissue density was calculated as the ratio of stem dry weight to fresh volume.

### 2.4 High-throughput sequencing of soil microorganisms

Total genomic DNA was extracted from 0.5 g of homogenized fresh soil using the FastDNA^®^ SPIN Kit (MP Biomedicals, USA) following the manufacturer’s protocol. The purity and concentration of the extracted DNA were assessed using a NanoDrop 2000 spectrophotometer (Thermo Fisher Scientific, USA), and qualified samples were subsequently processed for metagenomic sequencing. The amount of DNA extracted was measured using 1% agarose gel electrophoresis. Subsequent PCR amplification targeted the V3-V4 hypervariable region of bacterial 16S rRNA genes using universal primers 338F (5’-ACTCCTACGGGAGGCAGCAG-3’) and 806R (5’-GGACTACHVGGGTWTCTAAT-3’). The fungi were amplified using the ITS gene and universal primers ITS1F (5’-CTTGGTCATTTAGAGGAAGTAA-3’) and ITS2R (5’-GCTGCGTTCTTCATCGATGC-3’). The amplification protocol was performed as follows: initial denaturation at 95 °C for 3 min, followed by 27 cycles of amplification (denaturation at 95 °C for 30 s, annealing at 55 °C for 30 s, and extension at 72 °C for 30 s), with a final extension at 72 °C for 10 min. The amplified products were then maintained at 4 °C for storage (using an ABI GeneAmp^®^ 9700 thermal cycler). The PCR reaction was performed in a 20 μL system containing: 4 μL of 5× TransStart FastPfu buffer, 2 μL of 2.5 mM dNTPs, 0.8 μL each of forward and reverse primers (10 μM), 0.4 μL of TransStart FastPfu DNA polymerase (2.5 U/μL), and approximately 10 ng of template DNA. Each sample was amplified in triplicate. Following amplification, the triplicate PCR products for each sample were pooled and electrophoresed on a 2% agarose gel. Target DNA fragments were excised from the gel and purified using the AxyPrep DNA Gel Extraction Kit (Axygen Biosciences, Union City, CA, USA) according to the manufacturer’s instructions. PCR products were quantified using a Quantus™ Fluorometer (Promega, USA). Sequencing was performed using Illumina’s Miseq PE250 platform (Shanghai Meiji Biomedical Technology Co., LTD.).

### 2.5 Diversity measures

We quantified the three attributes of biodiversity, species diversity (SD), functional diversity (FD), and phylogenetic diversity (PD), using Hill-Chao numbers of order q = 1 (Chao et al., 2023; Li et al., 2024). The q = 1 Hill-Chao number assigns equal weight to both rare and common species, providing a more unbiased and accurate estimate of diversity. Under this framework, all three metrics share the same unit, thus enabling meaningful comparisons. Plant diversity was assessed across species diversity, phylogenetic diversity, and functional diversity using the “iNEXT” package in R 4.4.0. Functional diversity is quantitatively analyzed based on key traits closely related to water use, including specific leaf area, leaf thickness, root length, dry matter content, stem dry matter content, and stem tissue density. For phylogenetic diversity, species names were standardized according to the World Flora Online (https://www.worldfloraonline.org), and a phylogenetic tree was constructed to compute PD. Similarly, Hill-Chao numbers of order q = 1 were also applied to quantify soil bacterial diversity and fungal diversity.

### 2.6 Ecosystem multifunctionality

This study calculated plant productivity, carbon cycling, nitrogen cycling, and phosphorus cycling functions as proxies for multifunctionality. The average value method was commonly used to quantify EMF, due to its statistical robustness and ease of interpretation (Byrnes et al., 2014; Li et al., 2022). Specifically, the measured values of the characterization indicators were initially standardized to a scale of 0-1. Subsequently, a single function was represented by averaging multiple characterizing indicators. Finally, the multifunctionality was characterized by calculating the average of the four single function indices. In addition, the multi-threshold approach was applied to assess the link between species diversity and the number of functions exceeding critical thresholds (ranging from 5% to 99%), based on plant productivity, carbon cycling, nitrogen cycling, and phosphorus cycling functions (Byrnes et al., 2014), implemented using the “multifunc” package in R 4.4.0.

### 2.7 Statistical analysis

First, one-way analysis of variance (ANOVA) followed by Turkey’s test was used to examine differences in individual ecosystem function that conformed to a normal distribution. Second, Pearson correlation analyses were applied to assess the relationship among plant diversity, soil microbial diversity, abiotic factors, and EMF under changing rainfall patterns. Additionally, linear mixed models (LMM) were implemented using the “nlme” package in R 4.4.0 to evaluate how SWC, soil pH, rainfall frequency, plant diversity, and soil microbial influence EMF. In this model, SWC, soil pH, rainfall frequency, plant species diversity, plant functional diversity, plant phylogenetic diversity, soil bacterial diversity, and soil fungal diversity were treated as fixed effects, with the four rainfall treatments as random effects. Finally, based on the results of rainfall treatment and linear mixed models, six factors identified as drivers of EMF were selected to construct a piecewise structural equation model (SEM) using the “piecewiseSEM” package in R 4.4.0 (Lefcheck and Freckleton, 2015). In this model, SWC, pH, and rainfall frequency (RF) were included as abiotic factors, plant species diversity and functional diversity as plant factors, and soil bacterial diversity as the microbial factor. This SEM was used to analyze the mechanisms by which these factors influence EMF under changing rainfall patterns in a karst region. The model fit was assessed using Fisher’s C statistic, and this result indicates a non-significant *p*-value (*p* > 0.05), confirming that the model adequately fits the observed data. This threshold is consistent with standard SEM practices. All abbreviations in this article are listed in **Supplementary Table 3**.

## 3 Results

### 3.1 Biodiversity and ecosystem multifunctionality

**Figure 2:**
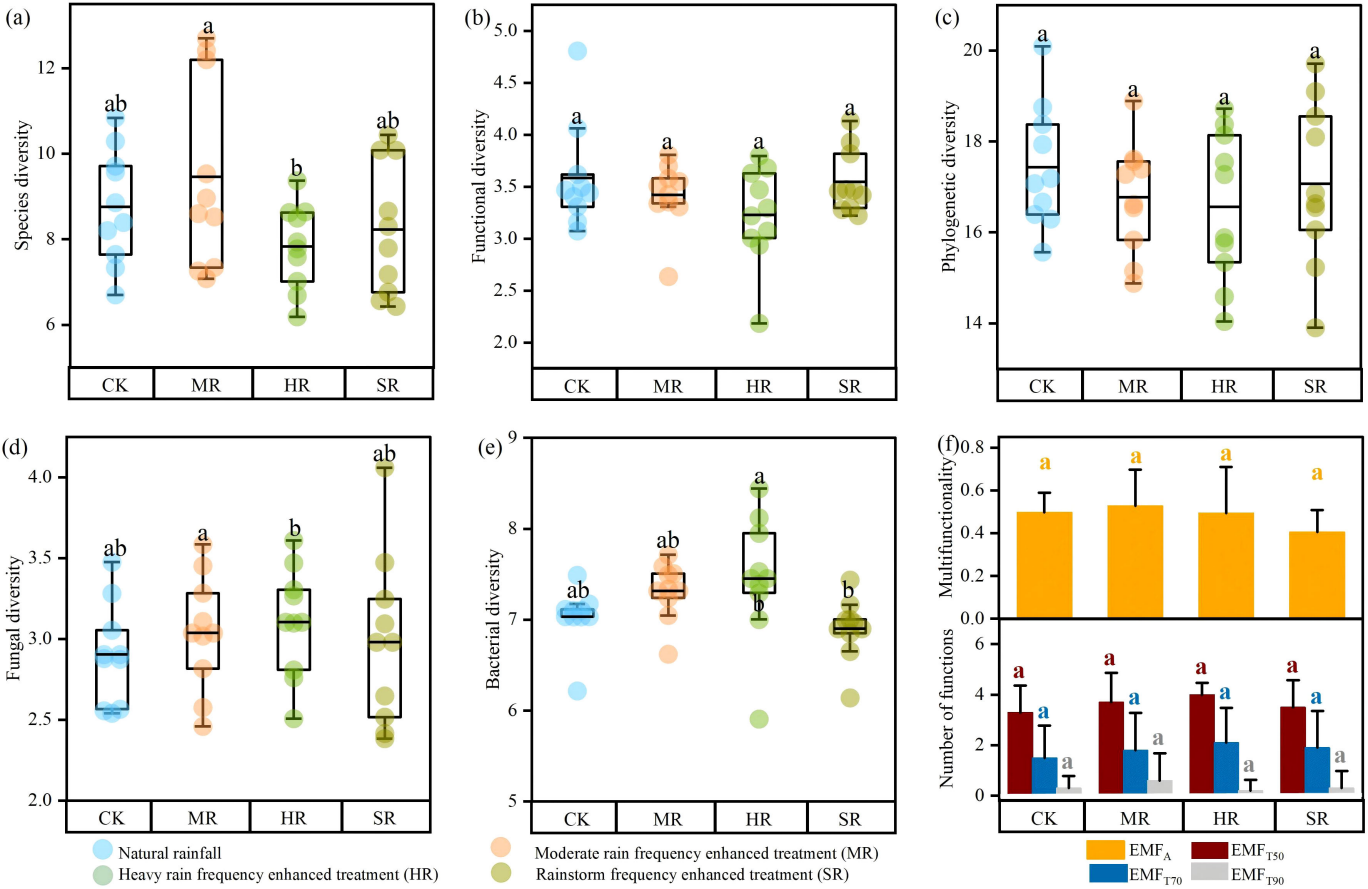
Effects of changing rainfall patterns on biodiversity and ecosystem multifunctionality, including plant species diversity **(a)**, plant functional diversity **(b)**, plant phylogenetic diversity **(c)**, soil fungal diversity **(d)**, soil bacterial diversity **(e)**, and ecosystem multifunctionality **(f)** under changing rainfall patterns in the initial stage of abandoned karst farmland. In a-e, the rectangles represent the 25th to 75th percentiles; the black horizontal lines symbolize the average values. The same colors show the same rainfall treatments. Different lowercase letters indicate statistically significant differences between treatments (*P* < 0.05). In f, the same letters in yellow, red, green, and gray, respectively, represent averaged and three threshold levels of multifunctionality, showing no significant differences among different treatments. EMF_A_: averaging-based multifunctionality; EMF_T50_, EMF_T70_, EMF_T90_: multifunctionality at 50%, 70% and 90% thresholds, respectively.

**Figure 3:**
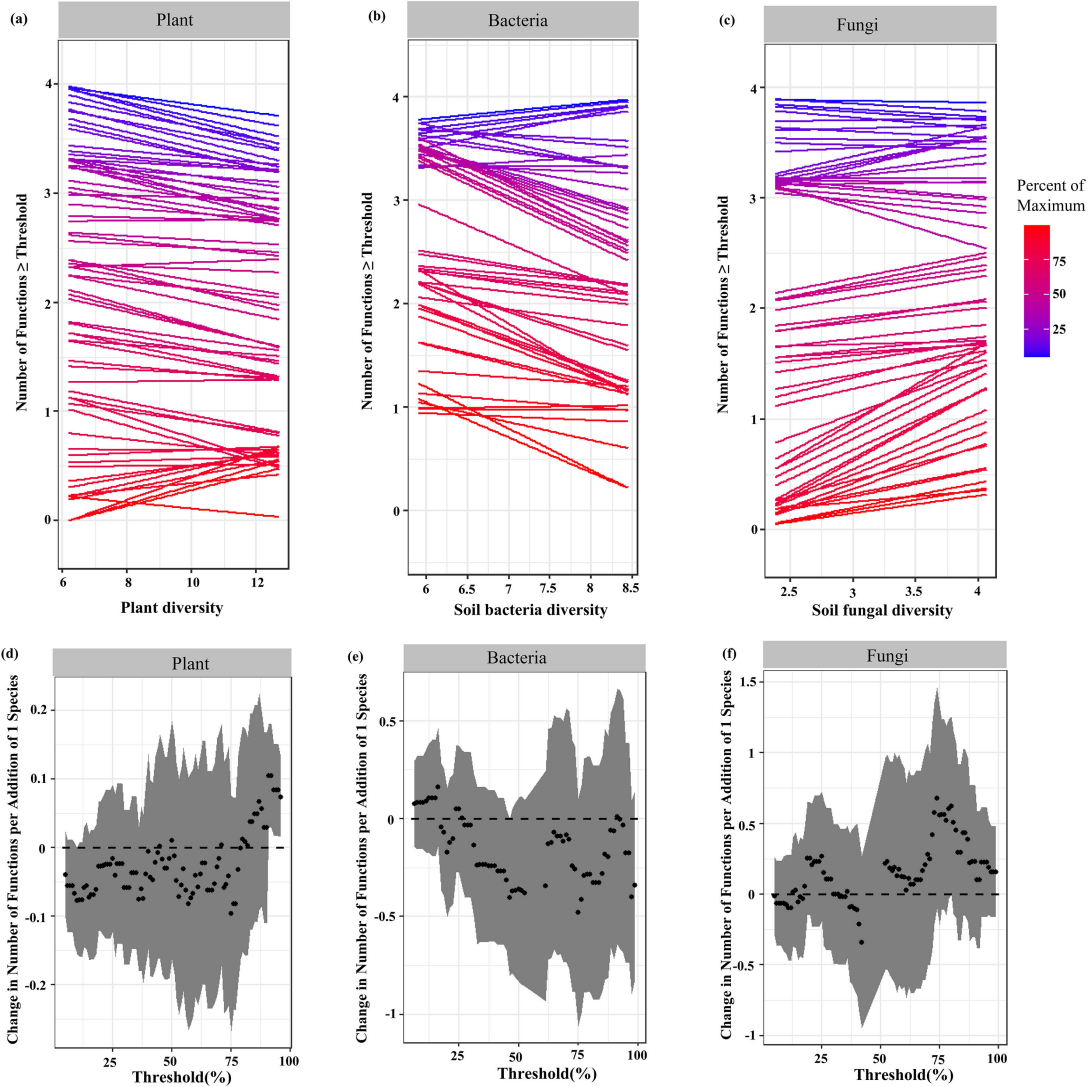
Relationship between plant **(a, d)**, bacterial **(b, e)** and fungal **(c, f)** diversity and simplified multiple-threshold multifunctionality.

Compared with the control treatment (CK), rainfall treatments reduced plant diversity but increased soil microbial diversity, though both effects were not statistically significant (*P* > 0.05; **Figure 2**). It is particularly noteworthy that, compared to the moderate rainfall frequency enhancement treatment (MR), the heavy rainfall frequency enhancement treatment (HR) significantly inhibited plant species diversity but significantly promoted soil fungal diversity (*P* < 0.05, **Figures 2a, d**). Compared to the rainstorm frequency enhancement treatment (SR), the HR treatment significantly enhanced soil bacterial diversity (*P* < 0.05, **Figure 2e**). Furthermore, the HR treatment promoted ecosystem multifunctionality (EMF) (*P* > 0.05; **Figure 2f**). Multi-threshold analysis revealed that the regulatory effects of plant, bacterial, and fungal diversity on EMF vary with thresholds (**Figure 3**). Plant diversity inhibited EMF below 40%, became positively influential above 40%, with the peak effect at 85%. Bacteria exerted an inhibitory effect on EMF, strongest at low to medium thresholds. Fungi began contributing positively beyond 55%, with the optimal range at 70% - 80%. The overall strength of these effects differed across thresholds: bacterial inhibition dominated low to medium thresholds, while at high thresholds the positive contributions ranked as plants > fungi > bacteria.

### 3.2 The relationship between abiotic factors, biotic factors, and ecosystem multifunctionality

**Figure 4:**
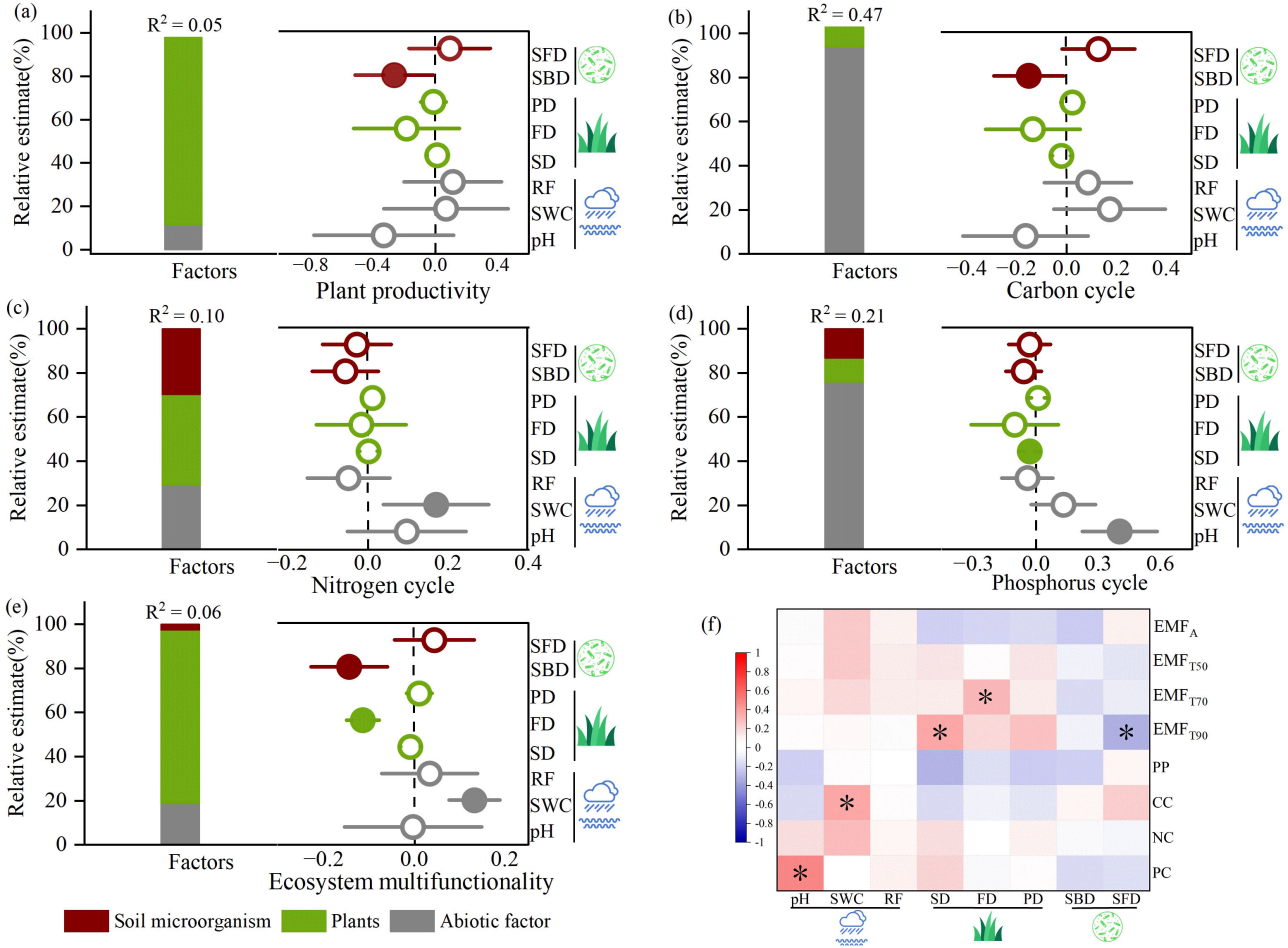
The relative effects of plants, soil microorganisms, and abiotic factors in the initial stage of abandoned karst farmland on plant productivity **(a)**, the carbon cycle **(b)**, the nitrogen cycle **(c)**, the phosphorus cycle **(d)** and ecosystem multifunctionality (calculated by the average value method) **(e)** and **(f)** the relationships between the three types of factors and single function and ecosystem multifunctionality. The model averages the relative importance of the three sets of explanatory variables (left), and the linear mixed-models parameters are estimated with normalized regression coefficients ± 95% CIs (right). The significance level is indicated by solid circles: *P* < 0.05. All statistical analyses are performed using a linear mixed model with rainfall treatment as the random factor. Red is an indicator of soil microbial diversity, green is an indicator of plant diversity, and gray is an indicator of abiotic factors. In the correlation figure, red indicates a positive correlation, and blue indicates a negative correlation. Significance levels are indicated by asterisks: *P < 0.05. pH, alkalinity; SWC, soil water content; RF, rainfall treatment; PD, plant phylogenetic diversity; FD, plant functional diversity; SD, plant species diversity; SBD, soil bacterial diversity; SFD, soil fungal diversity; EMFA, averaging-based multifunctionality; EMFT50, EMFT70, EMFT90, multifunctionality at 50%, 70% and 90% thresholds, respectively.

There were differences in the effects of abiotic factors and biotic factors on individual ecosystem function or multifunctionality (**Figure 4**; **Supplementary Figures 3**, **4**). Specifically, among biotic factors, soil bacterial diversity had a significant negative effect on plant productivity (PP) and EMF (*P* < 0.05; **Figures 4a, e**), while plant species diversity had a significant negative effect on phosphorus cycling (PC) (*P* < 0.05; **Figure 4d**). Furthermore, plant functional diversity had a significant negative impact on multifunctionality (*P* < 0.05; **Supplementary Figure 4**). Among abiotic factors, soil water content (SWC) showed a significant positive influence on nitrogen cycling (NC), EMF, and EMF_70_ (*P* < 0.05; **Figures 4c, e**), and soil pH was significantly positively associated with PC (*P* < 0.05; **Figure 4d**).

### 3.3 Drivers of ecosystem multifunctionality

**Figure 5:**
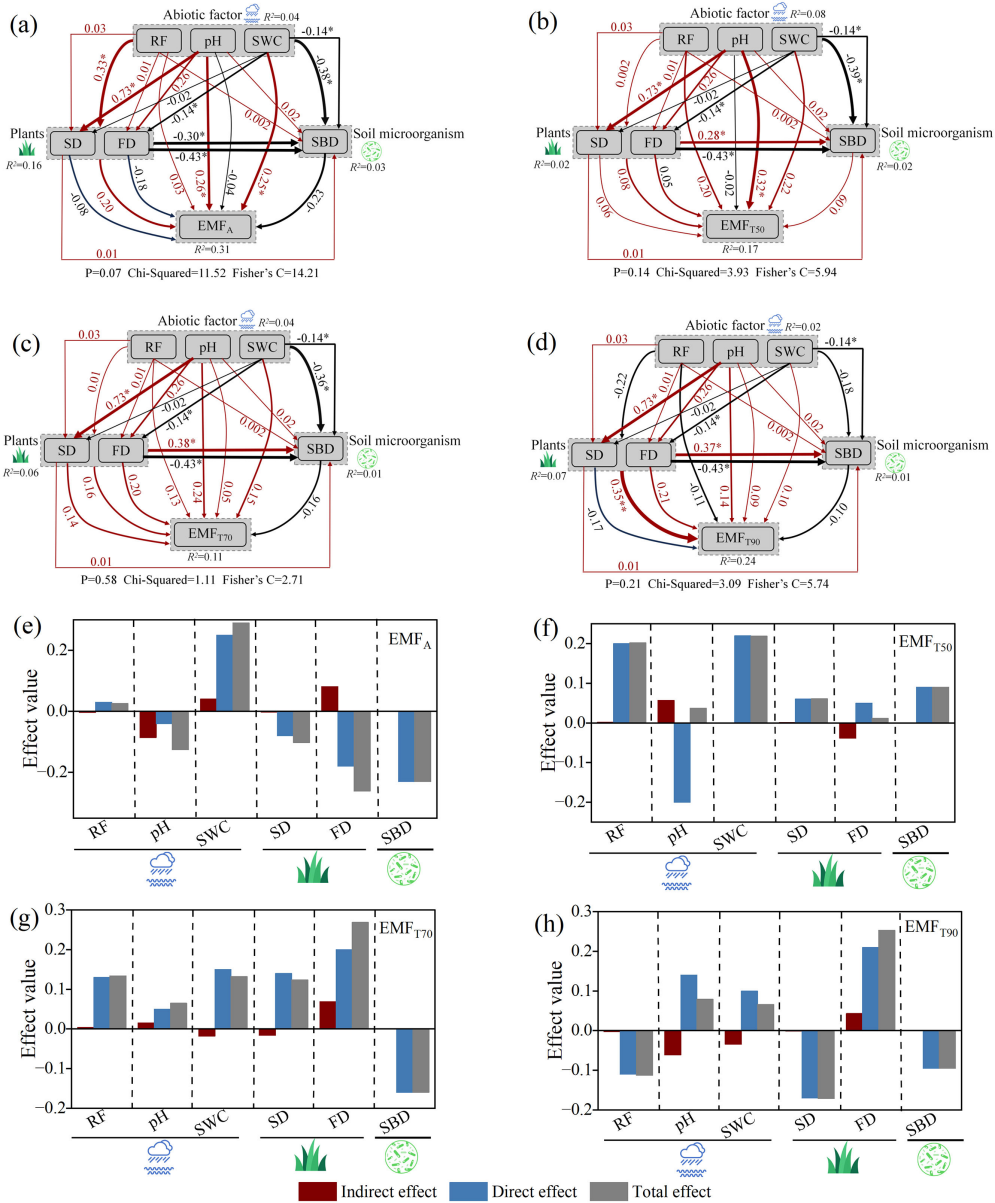
Piecewise structural equation models exploring the relationships between plant diversity, soil microbial diversity, abiotic factors, and averaged and three threshold levels of multifunctionality **(a–d)**. The red solid line arrows represent a positive correlation, the black solid line arrows represent a negative correlation. The line thickness represents the strength of the correlation. Significance levels are indicated by asterisks: *P < 0.05, ** *P* < 0.01. Effect sizes of plant diversity, soil microbial diversity, and abiotic factors on averaged and three threshold levels of multifunctionality **(e–h)**. Red bars represent indirect effects, blue bars represent direct effects, and gray bars represent the total effect. pH, acidity/alkalinity; SWC, soil water content; RF, rainfall treatment; FD, plant functional diversity; SD, plant species diversity; SBD, soil bacterial diversity; EMFA, averaging-based multifunctionality; EMFT50, EMFT70, EMFT90, multifunctionality at 50%, 70% and 90% thresholds, respectively.

As the multifunctionality screening threshold increased from EMF_T50_ to EMF_T90_, the primary drivers of EMF exhibited a gradient shift (**Figures 5a–h**). Regarding the path relationships, the overall plant community effect on soil bacterial diversity (SBD) shifted from a significant negative effect (-0.30*) under EMF_A_ to a consistently significant positive effect (0.28* - 0.38*) under thresholds from EMF_T50_ to EMF_T90_. At the component level, functional diversity (FD) directly and positively drove EMF across all thresholds, with a stronger effect at higher thresholds. Species diversity (SD) consistently exerted a negative effect on EMF across thresholds, intensifying with increasing thresholds. Abiotic factors exhibited significant positive direct effects at EMF_A_, which gradually declined with higher thresholds. Soil microorganisms overall played a negative regulatory role in EMF. Furthermore, the total effect of plants increased continuously with rising thresholds, supported by both the direct effect of FD and the plant-mediated indirect pathways via SBD. Under high-threshold conditions, plant functional diversity replaced abiotic factors as the primary driver regulating EMF.

## 4 Discussion

### 4.1 Impact of rainfall pattern changes on environmental factors and multifunctionality

Our results indicate that changes in rainfall patterns differentially affect aboveground and belowground biodiversity in abandoned karst farmland, only partially supporting our first hypothesis. Contrary to expectations that increased rainstorm frequency would universally suppress biodiversity, we observed that, compared to natural rainfall, the HR treatment did not significantly reduce plant species diversity but instead non-significantly increased the diversity of soil fungi and bacteria (**Figures 2**, **3**). These findings suggest a decoupling of plant and microbial responses to rainfall patterns, likely arising from their differential sensitivities to soil moisture dynamics and niche differentiation (Engelhardt et al., 2018). This decoupling can be attributed to their niche differentiation in heterogeneous karst habitats (Chen et al., 2018). Specifically, plants primarily occupy aboveground resource niches and respond directly to variations in light, water, and temperature (Zhang and Xi, 2021). In contrast, soil microorganisms are largely constrained by belowground resources, such as soil organic carbon availability and local microenvironmental nutrient conditions (Chen et al., 2026). Altered rainfall regimes directly affect plant water availability and photosynthetic carbon allocation, whereas their effects on microorganisms are primarily indirect, mediated by changes in the quality and quantity of plant-derived carbon inputs, a process that involves a substantial time lag (Xun et al., 2021; Wang et al., 2025). Notably, despite the negative effects of rainfall treatments on plants and the observed increase in soil microbial diversity, a high level of EMF was achieved under these conditions.

This study clearly demonstrated that the relative effects of abiotic and biotic factors on EMF differ, and that their relative importance varied with the multifunctionality threshold. Among abiotic factors, soil water content (SWC) consistently exerts a positive effect on nitrogen cycling and EMF, whereas soil pH significantly promotes phosphorus cycling. In contrast, the effects of biotic factors are context-dependent. Bacterial diversity negatively affects plant productivity and EMF. However, as the threshold increases from moderate to high levels (e.g., EMF_T90_), the effect of plant functional diversity on EMF shifts from negative to positive. This threshold-dependent effect reversal suggests a dynamic regulation mechanism underlying the cost-benefit balance of biotic factors (Anujan et al., 2021; Luo et al., 2022).

### 4.2 Impacts of abiotic factors on ecosystem multifunctionality

The influence of soil water changes on ecosystem functions is variable and complex (Peng et al., 2024; Zhong et al., 2025). A decrease in rainfall frequency leads to larger rainfall events and longer intervals of rainfall, resulting in soil waterlogging or water shortages, which causes greater fluctuations in soil water, and has either positive or negative impacts on ecosystem functions (Knapp et al., 2008; Yang et al., 2023). In xeric ecosystems, reduced precipitation frequency has been shown to enhance ecosystem functioning by mitigating water stress, consistent with the soil water bucket’ model proposed by Knapp et al. (2008). This model proposes that reduced rainfall frequency leads to larger individual precipitation events, enhances water infiltration depth, and reduces evaporative losses, which in turn increase SWC availability for plant uptake (Knapp et al., 2008; Heisler-White et al., 2009). The results also clearly demonstrated that, among abiotic factors, SWC exerted the strongest direct effect on EMF (**Figure 4e**). Water availability is a primary limiting factor for ecosystem function in arid, semi-arid, and karst regions. SWC directly regulates soil microbial activity, enzymatic reaction rates, nutrient dissolution and transport, as well as plant root water uptake and physiological processes, thereby synchronously influencing multiple functional processes such as carbon, nitrogen, and phosphorus cycling, along with overall multifunctionality (Tang et al., 2023).

Our study found that changing rainfall patterns did not significantly affect any single ecosystem function, yet they altered multi-threshold multifunctionality (**Figure 2**). This aligns with a core perspective in multifunctionality research that environmental changes may reconfigure trade-offs and synergies among multiple functions, generating significant effects at an integrated level that can remain obscured when examining individual function in isolation (Hector and Bagchi, 2007; Allan et al., 2015). Specifically, altered soil water content may asynchronously influence different processes such as carbon, nitrogen, and phosphorus cycling, with the net effect ultimately manifesting in the multifunctionality (Gamfeldt et al., 2008; Zavaleta et al., 2010). Furthermore, our findings revealed that the influence of abiotic factors on EMF exhibited pronounced “threshold-dependence” whereby their direct effects weakened as the functional threshold increased (**Figure 5**). This is consistent with the consensus in multifunctionality studies that the relationship between drivers and multifunctionality highly depends on the level of functional performance considered (Byrnes et al., 2014; Manning et al., 2018). At low thresholds, suitable abiotic conditions (e.g., water, pH) may provide the foundational “baseline” for maintaining multiple functions; at high thresholds, however, more efficient biological regulatory mechanisms become essential. Specifically, changing rainfall patterns act as a powerful “environmental filter” (Keddy, 1992), reshaping the functional composition of plant communities by modifying the spatiotemporal heterogeneity of soil moisture and thereby selecting for plant species with specific adaptive traits, such as deep root systems (Ackerly, 2004; Funk et al., 2016). This filtering process ultimately modulates multifunctionality at high thresholds.

### 4.3 Plant functional diversity had a stronger effect on ecosystem multifunctionality

Functional diversity, based on functional traits, has been found to be a better predictor of EMF than species diversity (Hu et al., 2022; Pichon et al., 2024). Our study confirmed that plant functional diversity was a stronger driver of EMF (**Figure 5**). Because there are differences in functional traits among different species, functional diversity is a synthesis of functional traits of different species (Sobral, 2023). Moreover, functional diversity reflects the strategies of plants to obtain resources from the environment (Petchey and Gaston, 2002). For example, indicators such as plant height and specific leaf area are closely related to the light acquisition strategies of plants. During the initial stage of succession in abandoned karst farmland, resource-acquisition plants dominated, characterized by tall plants with small specific leaf areas. These species altered the environment by changing rainfall patterns, providing a better physical basis for later species (Mudrák et al., 2016). In addition, this study found that functional diversity had a positive impact on EMF (**Figure 5**). This might be because niche complementarity increased functional diversity in the initial stages of abandoned karst farmlands. Higher functional diversity typically buffered the negative effects of water changes on EMF, thereby, increasing ecosystem resistance to water change and altering EMF (Ali et al., 2019).

Interestingly, it was noteworthy that as the multifunctionality screening threshold increased from EMF_t50_ to EMF_T90_, the primary drivers of EMF exhibited a gradient shift from abiotic factors to plant functional diversity. This result revealed that the regulatory mechanisms of EMF were not constant but are closely related to the degree of “high standards”. Specifically, the driving effect of abiotic factors was optimal under low-threshold conditions but progressively declined as the threshold increased, with both direct and indirect positive effects continuously attenuating. This indicated that the abiotic environment played a dominant role in supporting lower levels of EMF; however, when multiple functions were required to be simultaneously achieved at a high level, the constraining role of abiotic factors became relatively weaker (Yang et al., 2026). In contrast, plant functional diversity replaced the abiotic environment as the primary driver regulating EMF under high-threshold conditions. This shift relies on the mechanism of resource complementarity (Hu et al., 2021). Under high functional thresholds, ecosystems must synergistically sustain multiple high-level functions, which depends on the comprehensive and efficient use of multidimensional resources such as light, water, and nutrients (Byrnes et al., 2014; Yan et al., 2023). Plant communities with high functional diversity achieve niche differentiation and reduce interspecific competition through the complementarity of functional traits, thereby maximizing resource capture and utilization efficiency (van der Plas et al., 2016). This provided the foundational resource base for processes like carbon fixation and nutrient cycling to concurrently reach high levels (Mori et al., 2016). Second, in environments with highly fluctuating soil moisture (e.g., under extreme rainfall treatments), communities with high functional diversity were more likely to contain species with divergent stress-response strategies. When certain species experience functional decline due to water stress, other functional types can perform similar ecological roles (i.e., functional redundancy) and provide compensatory effects. This buffers the negative impact of environmental fluctuations on overall multifunctionality and maintains ecosystem stability at high thresholds (Isbell et al., 2015; Craven et al., 2016). Therefore, evaluating the impact of global extreme rainfall on EMF in abandoned karst farmland from the perspective of plant functional diversity, specifically elucidating its differential driving mechanisms across varying functional performance thresholds, represents a critical direction for future research.

Furthermore, soil microorganisms in this study exhibited an overall negative regulation of multifunctionality, with their total effect consistently remaining at a low level. This may be attributed to the strong coupling between microbial communities and environmental variables in ecosystems subjected to rainfall treatments (Li et al., 2024). Previous studies have shown that climatic factors are dominant drivers regulating microbial diversity and their functions, and that the contribution of microbial diversity to EMF largely depends on the synergistic effects of climatic background and soil environmental conditions (Chen et al., 2025). So, the direct effects of rainfall treatments weakened the functional contribution of microorganisms in this study.

We acknowledge certain limitations. For practical reasons, soil physicochemical and microbial variables were measured in four plots per treatment, and the remaining six plots per treatment were assigned the treatment-specific mean values, justified by the pre-experimental homogenization of the soil. This pragmatic approximation may underestimate within-treatment variance, but robustness checks showed that all detected effects exceeded the minimum detectable effect size (Cohen’s d > 2.38) given the sample size. Additionally, the experiment was conducted over a single year; the reported rainfall effects represent initial responses, and long-term monitoring is required to capture potential temporal dynamics. We therefore suggest that future studies incorporate larger soil sample sizes and multi-year observations to validate and extend our conclusions.

## 5 Conclusions

In conclusion, this study demonstrated that abiotic environmental factors can directly or indirectly affect the ecosystem multifunctionality of abandoned karst farmland. Among biotic factors, plant functional diversity served as a key mediating variable through which abiotic factors indirectly influence multifunctionality, and its relative importance increased with rising multifunctionality thresholds. Consequently, plant functional diversity was identified as a major predictor and driver of ecosystem multifunctionality in this system. These findings emphasized that restoration and management strategies for fragile karst ecosystems should shift from merely increasing species richness to proactively establishing and maintaining high plant functional diversity. In the process of vegetation restoration, priority should be given to assembling species with complementary key traits to build ecosystems with high functional redundancy, thereby enhancing their resilience to hydrological fluctuations and synergistically providing multiple high-level ecosystem functions. In addition, future long-term research with larger soil sample sizes is needed to validate and extend our conclusions.

## Data Availability

The original contributions presented in the study are included in the article/**Supplementary Material**. Further inquiries can be directed to the corresponding authors.
