# Supplementary material for: Plant functional diversity promotes ecosystem multifunctionality in abandoned karst farmland under changing rainfall patterns
Source: Front Plant Sci. 2026 Jul 13;17:1883399. doi: 10.3389/fpls.2026.1883399 (PMC13402464; doi:10.3389/fpls.2026.1883399)
Supplement: Supplementary file 1 [file DataSheet1.docx]

**Plant functional diversity promotes ecosystem multifunctionality in abandoned karst farmland under changing rainfall patterns**

Yuancai Qi ^a^, Maji Wan ^a^, Jinchun Liu ^a,b^, Shehzadi Kiran ^a^, Weixue Luo ^a,b^ *, Jianping Tao ^a,b^ *

^a^ Key Laboratory of Eco-environments in the Three Gorges Reservoir Region (Ministry of Education), School of Life Sciences, Southwest University, Chongqing 400715, China

^b^ Chongqing Jinfo Mountain Karst Ecosystem National Observation and Research Station, Southwest University, Chongqing 400715, China

**Supplementary Information**

This file includes:

Appendix S1: Index determination method

Appendix S2: Fig. S1 to S4

Appendix S3: Table S1 to S3

**Appendix S1: Index determination method**

**1.1 Measurement methods of ecosystem single function**

1. **Plant productivity**

**Aboveground biomass (AGB)**: We collected the aboveground part with an area of 0.25 m × 0.25 m, put it in an oven at a temperature of 80 ℃, dried to constant weight, weighed the aboveground part as aboveground biomass, then estimated the aboveground biomass of the whole community.

**Belowground biomass (BGB)**:We collected the underground part with an area of 0.25m×0.25m, put it into an oven at a temperature of 80 ℃, dried it to constant weight, and weighed the dried underground part as the underground biomass. Then we estimated the underground biomass of the whole community.

1. **Carbon cycle**

**Soil organic carbon (SOC)**: The potassium dichromate oxidation method was used (Song et al., 2022). The specific operations demonstrate: soil organic carbon (SOC) was titrated against a 0.5 M ferrous iron solution after it had been digested with 0.8 M K_2_Cr_2_O_4_ and concentrated H_2_SO_4_ at 150°C for 30 min.

**Microbial biomass carbon (MBC)**: The chloroform fumigation-potassium sulfate extraction method was used for the determination (Oberson et al., 1997). The specific operations demonstrate: took two parts of 10 g fresh soil and placed them in a conical bottle; one part of it was placed in a vacuum drying oven at 25℃ and shielded from light for 24 hours; three small beakers (one was loaded with CHCl_3_, some glass beads were placed in the bottom of the cup for explosion-proof, one was loaded with dilute NaOH solution, and one was placed in ultrapure water). After 24 h, the fumigated soil sample was removed and was added to 40 ml of 0.5 mol/L K₂SO₄ solution. It was shaken at room temperature for 30 min, then filtered. The extract was determined using a TOC-L_CPH_ automatic analyzer (Shimadsu, Japan).

**Soil sucrase (S-SC)**: Soil sucrase was determined by 3,5-dinitrosalicylic acid colorimetry (Yang et al., 2020).

1. **Nitrogen cycle**

**Soil total nitrogen (TN)**: An Elementar Vario EL cube was used (Weixian et al., 2003). The specific operations demonstrate: weighed 40 mg of an air-dried soil sample after 100 mesh sieve in tin foil, determined the soil total nitrogen content with an elemental analyzer (Elementar Vario).

**Inorganic nitrogen (IN)**: The content of soil inorganic nitrogen was the sum of ammonia nitrogen and nitrate nitrogen.

**Nitrate nitrogen (NO_3_^-^-N)**: Ultraviolet spectrophotometry was used (Weixian et al., 2003). The specific operations demonstrate: weighed 10 g fresh soil into a conical bottle, added 50 mL KCl solution, shook and filtered, absorbed the filtrate, added 1 mol/L HCl solution, then set the volume, and compared the color after 1 h.

**Ammonium nitrogen (NH_4_^+^-N)**: Indophenol blue colorimetry was used (Lu et al., 2004). The specific operations demonstrate: weigh 10 g of fresh soil into a conical bottle, add 50 mL of KCl solution, shake, and filter; absorb the filtrate, dilute with water, add 5 mL of C6H6O solution and 5 mL of NaClO solution, shake, leave at room temperature for 1 h, add 1 mL of a mask agent, then set the volume, and compare the color after 2 h.

**Microbial biomass nitrogen (MBN)**: The chloroform fumigation-potassium sulfate extraction method was used for the determination (Oberson et al., 1997). The specific operations demonstrate: weighed 10 g of fresh soil in a triangular bottle, put it in a vacuum drying oven, placed a beaker containing 200 mL chloroform, added 100 mL dilute NaOH solution, and H_2_O. The fumigation time was 24 h. In addition, the same amount of fresh soil was weighed and placed in another drying oven for non-fumigation control. After 24 h, let off the gas, remove the beaker containing chloroform, and repeatedly vacuum 4-5 times for 3 min each time. Added 25mL K_2_SO_4_ solution to the above two soil samples, shook for 30 min, then filtered. The content of soil microbial nitrogen was determined by the TOC-V_CPH_ organic carbon analyzer.

**Alkali-hydrolyzable nitrogen (AN)**: The alkaline hydrolysis diffusion method was used. The specific operations demonstrate: weighed 2 g of an air-dried soil sample through a 100-mesh sieve and 0.2 g of ferrous sulfate powder evenly spread in the outer chamber of the diffusion dish, and gently rotated the diffusion dish horizontally to smooth the soil sample. In the inner chamber of the diffusion dish, added 2 ml of a 2% boric acid solution containing an indicator, then coated the outer chamber edge of the dish with alkaline glycerin, covered the ground glass, and rotated it to make the ground glass completely bonded with the edge of the diffused blood, then slowly turned one side of the ground glass to expose a slit in the diffusion dish. Quickly added 10 mL of 1.07 mo1/L NaOH into the outer chamber of the diffusion dish, immediately rotated the ground glass tightly, gently rotated the diffusion dish horizontally on the test bench to mix the solution with the soil fully, and secured it with a rubber band. Then carefully placed in an incubator at 40 °C. After 24 h, the ammonia absorbed by boric acid solution in the diffusion pan chamber was determined with 0.005 mo1/L H_2_SO_4_ standardized solution drop using a microburette. Took another diffusion dish and made a control test without adding soil, the other steps were the same as those with soil.

1. **Phosphorus cycle**

**Soil total phosphorus (TP)**: The Elementar Vario EL cube is used (Weixian et al., 2003). The specific operations demonstrate: weighed 40 mg of an air-dried soil sample after a 100-mesh sieve in tin foil, determined the total phosphorus content of the soil with an elemental analyzer (Elementar Vario EL cube).

**Microbial biomass phosphorus (MBP)**: Chloroform was used for the determination (Oberson et al., 1997). The specific operations demonstrate: weighed 5 g of an air-dried soil sample after a 100-mesh sieve, put it in the dryer, and fumigated it with ethanol-free chloroform. An equal amount of soil was placed in another dryer, not fumigated, as a control. After 24 hours, the fumigated and unfumigated soil were transferred to a 250 ml plastic bottle, added 100 mL of 0.5 mol/L NaHCO_3_ solution, shaken at 25℃ and 250 r/min for 30 min, and filtered with a slow qualitative filter paper. The content of inorganic phosphorus was determined by molybdenum-antimony resistance colorimetry. The following formula was used to calculate soil microbial biomass phosphorus:

$$\text{MBP}=\text{ }\frac{\text{EP}_{\text{i}}}{\text{k}_{\text{p}}}$$

Where, EP_i_ was the difference between fumigated soil and unfumigated soil; k_p_ indicated the conversion coefficient, and its value was 0.4.

**Acid phosphatase (ACP)**: An ultraviolet spectrophotometer method was used (Lu et al., 1999). The specific operations demonstrate: weighed 1 g of air-dried soil after 100 mesh sieve into a 50 mL triangle bottle, added 0.2 mL toluene, 4 mL sodium acetate buffer (pH = 6.5, 0.2 mol/L), and 1 mL disodium p-nitrobenzene phosphate solution (10 mmol/L). Shook and placed in a constant temperature incubator at 37 ºC for 1 h. Immediately added 1mL CaCl_2_ and 4 mL NaOH, shook, and filtered with qualitative filter paper. Took the filtrate for colorimetric determination at 420 nm on a UV-visible spectrophotometer. Phosphatase activity was expressed as the mass of p-nitrophenol released per gram of soil per unit time.

**Phosphodiesterases (PDEs)**: An ultraviolet spectrophotometer method was used (Lu et al., 1999). The specific operations demonstrate: weighed 1 g of air-dried soil after 100 mesh sieve into a 50 mL triangular bottle, added 0.2 mL toluene, 4 mL THAM-H_2_SO_4_ buffer (pH=8, 0.05 mol/L), and 1 mL bis-p-nitrophenyl phosphate (BPNP, 5 mmol/L). Shook and put into a constant temperature incubator at 37 ºC for 1 h. Immediately added 1 mL 0.5 mol/L CaCl_2_ and 4 mL THAM-NaOH, shook gently, filtered with qualitative filter paper into a centrifugal tube, and took the filtrate for colorimetric determination at 420 nm on a spectrophotometer. Phosphodiesterase activity was expressed as the mass of p-nitrophenol released per gram of soil per unit time.

**Residual phosphorus (RP)**: A colorimetric method was used (Lu et al., 1999). The specific operations demonstrate: took 0.5 g of air-dried soil after a 100-mesh sieve, added anion exchange resin, 0.5 mol /L NaHCO_3_, 0.1 mol/L NaOH, and 1 mol/L HCl step by step to extract soil phosphorus components with stability from weak to strong. Finally, concentrated nitric acid, hydrogen peroxide, and hydrofluoric acid were added to the microwave digester for high-temperature digestion, and the content of residual phosphorus was determined by molybdenum-antimony resistance colorimetry.

**Appendix S2: Fig. S1 to S5**

**
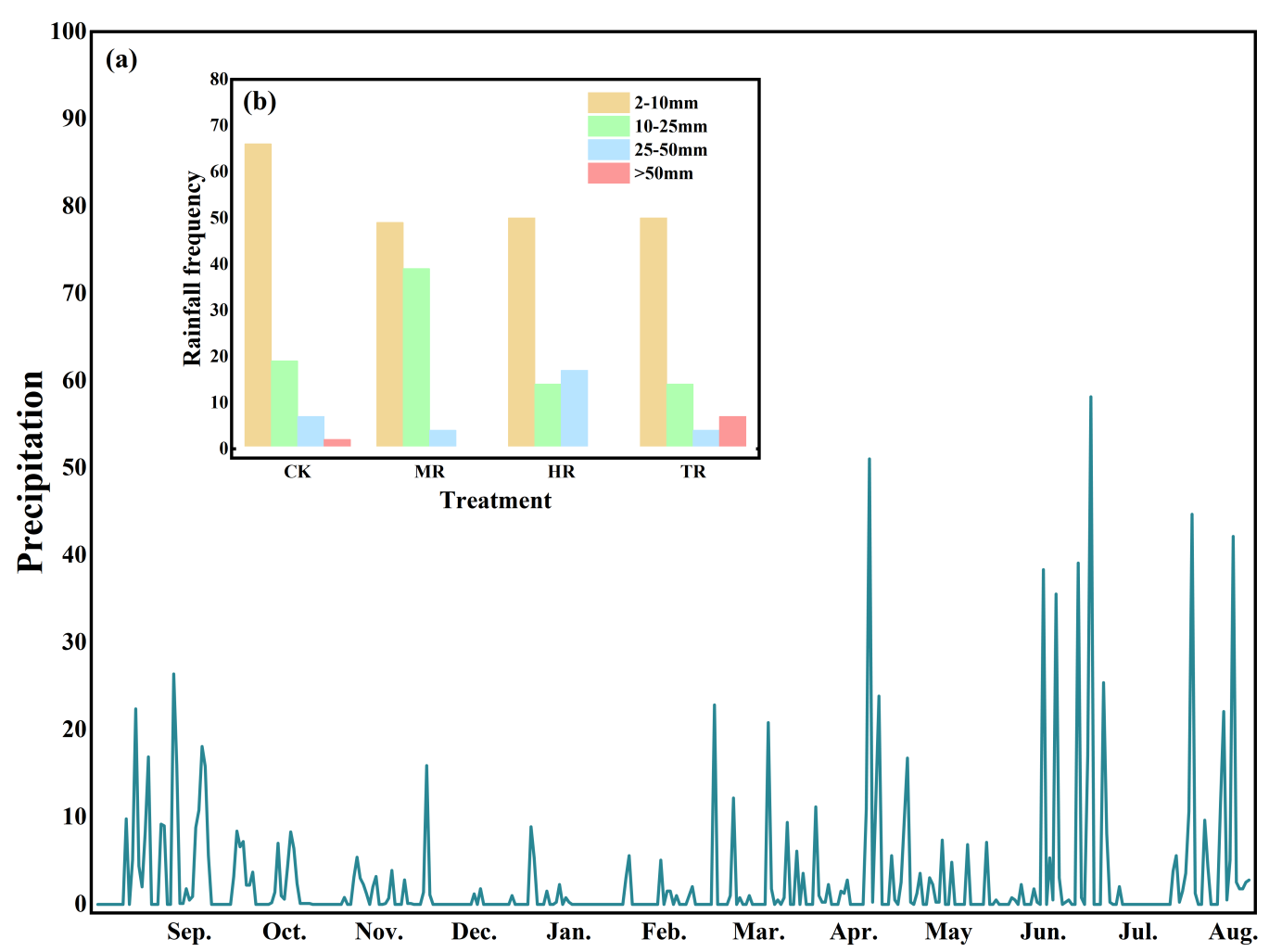
**

Fig.S1. Changes in natural rainfall and different rainfall intensification treatments. (a) is the natural rainfall data from September 2020 to August 2021. The blue curve shows rainfall data from September 2020 to August 2021, the first year of processing changes in rainfall patterns. (b) is the rainfall frequency during September 2020 to August 2021 in the different rainfall intensity frequency under each rainfall intensity treatment.


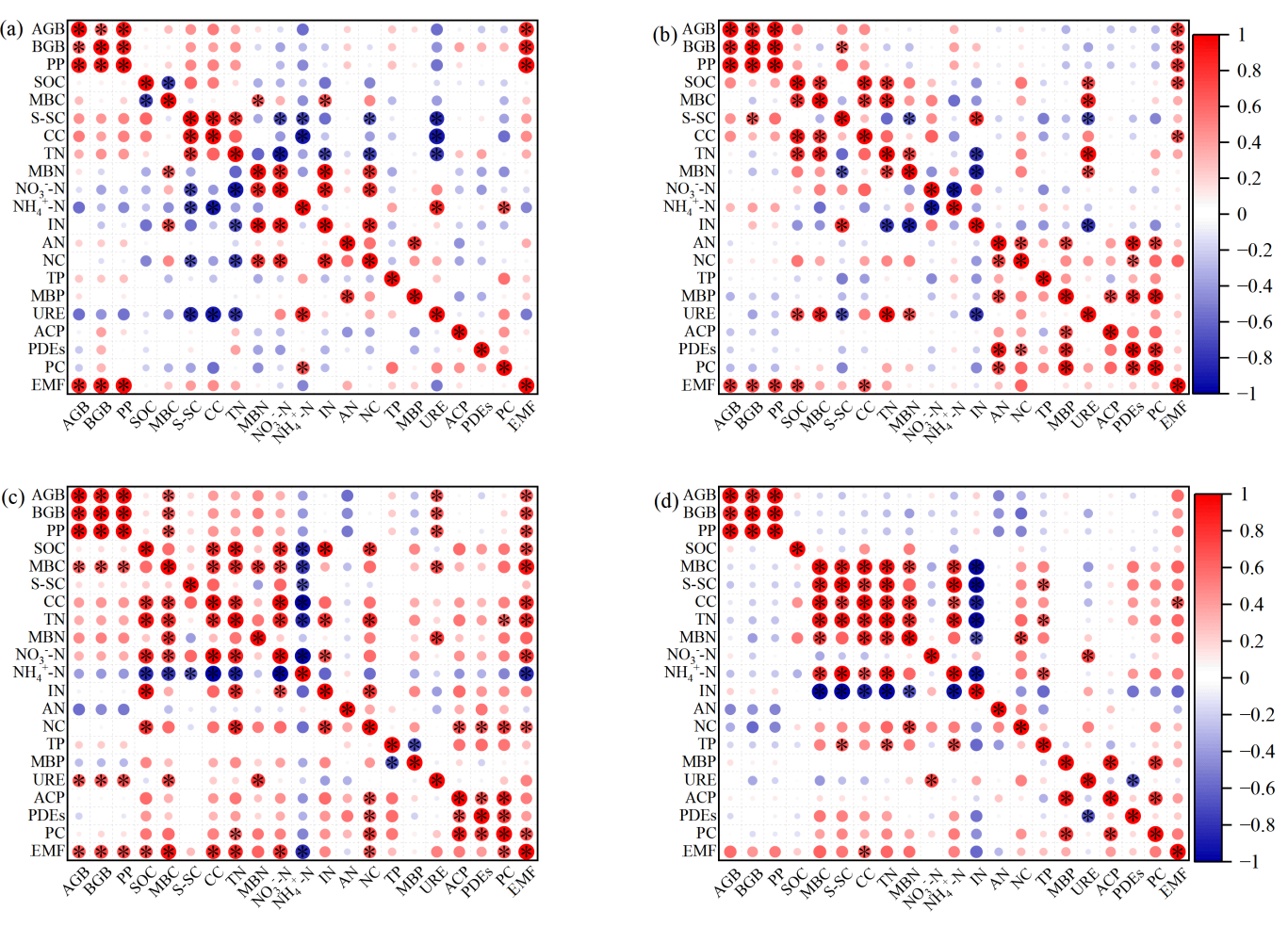


Fig.S2. Pearson correlation between single ecosystem function and multifunctionality (calculated by the average value method) in the initial stage of karst abandoned farmland under natural rainfall treatment (a), moderate rain frequency enhanced treatment (b), heavy rain frequency enhanced treatment (c), and rainstorm frequency enhanced treatment (d). Red represents a positive correlation, blue represents a negative correlation. Significance levels are indicated by asterisks: * *P* < 0.05. AGB: aboveground biomass; BGB: belowground biomass; PP: plant productivity; SOC: soil organic matter; MBC: soil microbial biomass carbon; S-SC: soil sucrase; CC: carbon cycling; TN: total nitrogen content; MBN: soil microbial biomass nitrogen content; NO3--N: nitrate nitrogen; NH4--N: ammonium nitrogen; IN: inorganic nitrogen; AN: alkali-hydrolyzable nitrogen; NC: nitrogen cycling; TP: total phosphorus content; MBP: soil microbial biomass phosphorus content; URE: urease; ACP: acid phosphatase; PDEs: phosphodiesterase; PC: phosphorus cycling; EMF: ecosystem multifunctionality.


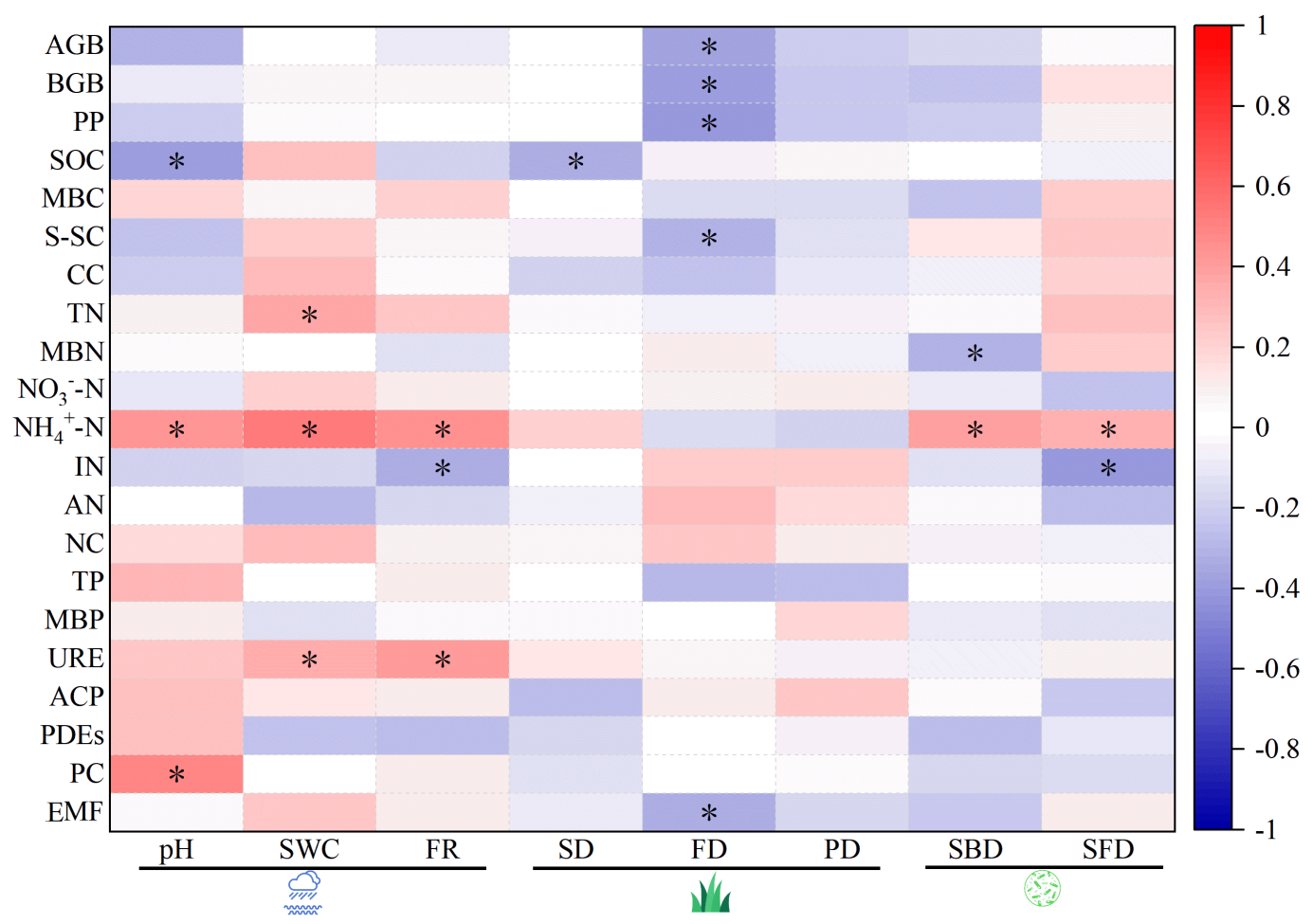


Fig.S3. Pearson correlation of plant diversity, soil microbial diversity, abiotic factors, single ecosystem function, and multifunctionality (calculated by the average value method). Red represents a positive correlation, blue represents a negative correlation. Significance levels are indicated by asterisks: * *P* < 0.05. pH: acidity/alkalinity; SWC: soil water content; RF: rainfall treatment; FD: plant functional diversity; SD: plant species diversity; SBD: soil bacterial diversity; AGB: aboveground biomass; BGB: belowground biomass; PP: plant productivity; SOC: soil organic matter; MBC: soil microbial biomass carbon; S-SC: soil sucrase; CC: carbon cycling; TN: total nitrogen content; MBN: soil microbial biomass nitrogen content; NO3--N: nitrate nitrogen; NH4--N: ammonium nitrogen; IN: inorganic nitrogen; AN: alkali-hydrolyzable nitrogen; NC: nitrogen cycling; TP: total phosphorus content; MBP: soil microbial biomass phosphorus content; URE: urease; ACP: acid phosphatase; PDEs: phosphodiesterase; PC: phosphorus cycling; EMF: ecosystem multifunctionality.


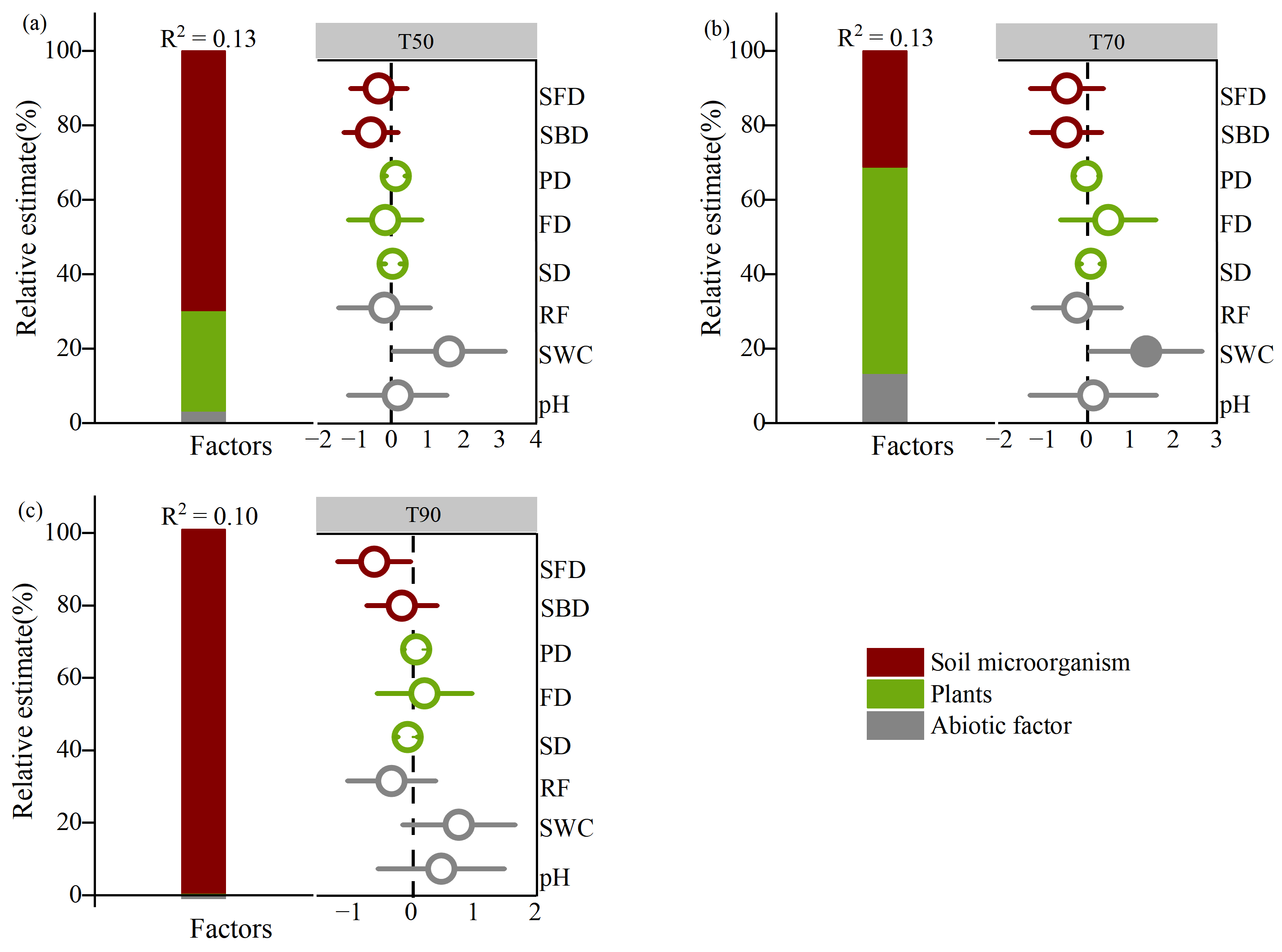


Fig.S4 The relative effects of plants, soil microorganisms, and abiotic factors in the initial stage of abandoned karst farmland on multifunctionality at the threshold 50 level (a), threshold 70 level (b), and threshold 90 level (c). The model averages the relative importance of the three sets of explanatory variables (left), and the linear mixed models parameters are estimated with normalized regression coefficients ± 95% CIs (right). The significance level is indicated by solid circles: *P* < 0.05. All statistical analyses are performed using a linear mixed model with rainfall treatment as the random factor. Red is an indicator of soil microbial diversity, green is an indicator of plant diversity, and gray is an indicator of abiotic factors. pH: acidity/alkalinity; SWC: soil water content; RF: rainfall treatment; PD: plant phylogenetic diversity; FD: plant functional diversity; SD: plant species diversity; SBD: soil bacterial diversity; SFD: soil fungal diversity; EMF_A_: averaging‑based multifunctionality; EMF_T50_, EMF_T70_, EMF_T90_: multifunctionality at 50%, 70% and 90% thresholds, respectively.

**Appendix S3: Table S1 to S3**

Table S1 Plant species under different rainfall patterns

| No. | Species | Families | Genus | Importance value (IV) | | | |
| --- | --- | --- | --- | --- | --- | --- | --- |
|  |  |  |  | CK | MR | HR | SR |
| 1 | *Erigeron canadensis* | Asteraceae | Erigeron | 4.09 | 3.21 | 3.20 | 3.41 |
| 2 | *Setaria viridis* | Poaceae | Setaria | 0.93 | 1.14 | 1.51 | 1.47 |
| 3 | *Oxalis corniculata* | Oxalidaceae | Oxalis | 0.75 | 0.56 | 0.48 | 0.58 |
| 4 | *Aster tataricus* | Asteraceae | Aster | 0.11 | 0.42 | 0.25 | 0.51 |
| 5 | *Duchesnea indica* | Rosaceae | Duchesnea | 0.27 | 0.38 | 0.72 | 0.48 |
| 6 | *Persicaria posumbu* | Polygonaceae | Persicaria | 0.55 | 0.47 | 0.69 | 0.46 |
| 7 | *Pteridium aquilinum var. latiusculum* | Dennstaedtiaceae | Pteridium | 0.28 | 0.67 | 0.48 | 0.35 |
| 8 | *Imperata cylindrica* | Poaceae | Imperata | 0.08 | 0.11 | 0.03 | 0.29 |
| 9 | *Clinopodium chinense* | Lamiaceae | Clinopodium | 0.13 | 0.17 | 0.09 | 0.26 |
| 10 | *Carota* | Apiaceae | Daucus | 0.06 | 0.42 | 0.39 | 0.25 |
| 11 | *Arthraxon hispidus* | Poaceae | Arthraxon | 0.19 | 0.55 | 0.20 | 0.25 |
| 12 | *Alternanthera philoxeroides* | Amaranthaceae | Alternanthera | 0.33 | 0.28 | 0.14 | 0.24 |
| 13 | *Pyracantha fortuneana* | Rosaceae | Pyracantha | 0.10 | 0.12 | 0.11 | 0.16 |
| 14 | [*Causonis japonica*](http://www.iplant.cn/info/Causonis%20japonica) | Vitaceae | [Causonis](http://www.iplant.cn/info/Causonis) | 0.05 | 0.08 | 0.08 | 0.12 |
| 15 | [*Aster indicus*](http://www.iplant.cn/info/Aster%20indicus) | [Asteraceae](http://www.iplant.cn/info/Asteraceae) | [Aster](http://www.iplant.cn/info/Aster) | 0.22 | 0.08 | - | 0.09 |
| 16 | [*Persicaria perfoliata*](http://www.iplant.cn/info/Persicaria%20perfoliata) | [Polygonaceae](http://www.iplant.cn/info/Polygonaceae) | [Persicaria](http://www.iplant.cn/info/Persicaria) | 0.15 | 0.02 | - | 0.07 |
| 17 | [*Lygodium japonicum*](http://www.iplant.cn/info/Lygodium%20japonicum) | [Lygodiaceae](http://www.iplant.cn/info/Lygodiaceae) | [Lygodium](http://www.iplant.cn/info/Lygodium) | 0.17 | 0.10 | 0.15 | 0.07 |
| 18 | [*Dichrocephala benthamii*](http://www.iplant.cn/info/Dichrocephala%20benthamii) | [Asteraceae](http://www.iplant.cn/info/Asteraceae) | [Dichrocephala](http://www.iplant.cn/info/Dichrocephala) | 0.15 | - | - | 0.07 |
| 19 | [*Solanum nigrum*](http://www.iplant.cn/info/Solanum%20nigrum) | [Solanaceae](http://www.iplant.cn/info/Solanaceae) | [Solanum](http://www.iplant.cn/info/Solanum) | 0.14 | 0.11 | 0.05 | 0.07 |
| 20 | [*Lactuca indica*](http://www.iplant.cn/info/Lactuca%20indica) | [Asteraceae](http://www.iplant.cn/info/Asteraceae) | [Lactuca](http://www.iplant.cn/info/Lactuca) | 0.04 | 0.02 | 0.02 | 0.06 |
| 21 | [*Buddleja asiatica*](http://www.iplant.cn/info/Buddleja%20asiatica) | [Scrophulariaceae](http://www.iplant.cn/info/Scrophulariaceae) | [Buddleja](http://www.iplant.cn/info/Buddleja) | - | 0.15 | 0.06 | 0.06 |
| 22 | [*Rubus parvifolius*](http://www.iplant.cn/info/Rubus%20parvifolius) | [Rosaceae](http://www.iplant.cn/info/Rosaceae) | [Rubus](http://www.iplant.cn/info/Rubus) | - | 0.33 | 0.18 | 0.06 |
| 23 | [*Xanthium strumarium*](http://www.iplant.cn/info/Xanthium%20strumarium) | [Asteraceae](http://www.iplant.cn/info/Asteraceae) | [Xanthium](http://www.iplant.cn/info/Xanthium) | - | 0.06 | 0.03 | 0.06 |
| 24 | [*Rubus coreanus*](http://www.iplant.cn/info/Rubus%20coreanus) | [Rosaceae](http://www.iplant.cn/info/Rosaceae) | [Rubus](http://www.iplant.cn/info/Rubus) | - | - | - | 0.06 |
| 25 | [*Lactuca sibirica*](http://www.iplant.cn/info/Lactuca%20sibirica) | [Asteraceae](http://www.iplant.cn/info/Asteraceae) | [Lactuca](http://www.iplant.cn/info/Lactuca) | 0.03 | 0.05 | 0.09 | 0.05 |
| 26 | [*Ixeris polycephala*](http://www.iplant.cn/info/Ixeris%20polycephala) | [Asteraceae](http://www.iplant.cn/info/Asteraceae) | [Ixeris](http://www.iplant.cn/info/Ixeris) | - | 0.03 | 0.08 | 0.05 |
| 27 | [*Commelina communis*](http://www.iplant.cn/info/Commelina%20communis) | [Commelinaceae](http://www.iplant.cn/info/Commelinaceae) | [Commelina](http://www.iplant.cn/info/Commelina) | 0.02 | 0.24 | 0.04 | 0.05 |
| 28 | [*Senecio scandens*](http://www.iplant.cn/info/Senecio%20scandens) | [Asteraceae](http://www.iplant.cn/info/Asteraceae) | [Senecio](http://www.iplant.cn/info/Senecio) | 0.05 | - | 0.02 | 0.05 |
| 29 | *Cyperus glomeratus L.* | Cyperaceae | Cyperus | 0.06 | - | 0.02 | 0.04 |
| 30 | *Rubus pinfaensis* | Rosaceae | Rubus | 0.03 | 0.03 | 0.22 | 0.03 |
| 31 | *Paederia foetida* | Rubiaceae | Paederia | 0.01 | - | - | 0.03 |
| 32 | *Ophiopogon japonicus* | [Liliaceae](https://www.plantplus.cn/info/Liliaceae?t=z) | [Ophiopogon](https://www.plantplus.cn/info/Ophiopogon?t=z) | - | 0.01 | - | 0.03 |
| 33 | [*Euryops pectinatus*](https://www.iplant.cn/info/Euryops%20pectinatus) | [Asteraceae](https://www.iplant.cn/info/Asteraceae) | [Euryops](https://www.iplant.cn/info/Euryops) | - | - | - | 0.03 |
| 34 | [*Celtis sinensis*](https://www.iplant.cn/info/Celtis%20sinensis) | [Cannabaceae](https://www.iplant.cn/info/Cannabaceae) | [Celtis](https://www.iplant.cn/info/Celtis) | 0.02 | - | 0.06 | 0.03 |
| 35 | [*Camphora officinarum*](https://www.iplant.cn/info/Camphora%20officinarum) | [Lauraceae](https://www.iplant.cn/info/Lauraceae) | [Camphora](https://www.iplant.cn/info/Camphora) | 0.04 | 0.07 | 0.17 | 0.02 |
| 36 | [*Justicia procumbens*](https://www.iplant.cn/info/Justicia%20procumbens) | [Acanthaceae](https://www.iplant.cn/info/Acanthaceae) | [Justicia](https://www.iplant.cn/info/Justicia) | 0.03 | 0.01 | 0.01 | 0.02 |
| 37 | [*Sedum sarmentosum*](https://www.iplant.cn/info/Sedum%20sarmentosum) | [Crassulaceae](https://www.iplant.cn/info/Crassulaceae) | [Sedum](https://www.iplant.cn/info/Sedum) | - | - | - | 0.02 |
| 38 | [*Stellaria aquatica*](https://www.iplant.cn/info/Stellaria%20aquatica) | [Caryophyllaceae](https://www.iplant.cn/info/Caryophyllaceae) | [Stellaria](https://www.iplant.cn/info/Stellaria) | - | - | - | 0.01 |
| 39 | [*Artemisia caruifolia*](https://www.iplant.cn/info/Artemisia%20caruifolia) | [Asteraceae](https://www.iplant.cn/info/Asteraceae) | [Artemisia](https://www.iplant.cn/info/Artemisia) | 0.07 | 0.01 | 0.14 | 0.01 |
| 40 | [*Viburnum dilatatum*](https://www.iplant.cn/info/Viburnum%20dilatatum) | [Viburnaceae](https://www.iplant.cn/info/Viburnaceae) | [Viburnum](https://www.iplant.cn/info/Viburnum) | - | - | - | 0.01 |
| 41 | [*Morus alba*](https://www.iplant.cn/info/Morus%20alba) | [Moraceae](https://www.iplant.cn/info/Moraceae) | [Morus](https://www.iplant.cn/info/Morus) | - | - | - | 0.01 |
| 42 | [*Cynodon dactylon*](https://www.iplant.cn/info/Cynodon%20dactylon) | [Poaceae](https://www.iplant.cn/info/Poaceae) | [Cynodon](https://www.iplant.cn/info/Cynodon) | 0.12 | - | 0.04 | - |
| 43 | [*Rubus idaeus*](https://www.iplant.cn/info/Rubus%20idaeus) | [Rosaceae](https://www.iplant.cn/info/Rosaceae) | [Rubus](https://www.iplant.cn/info/Rubus) | 0.12 | - | - | - |
| 44 | [*Oplismenus undulatifolius*](https://www.iplant.cn/info/Oplismenus%20undulatifolius) | [Poaceae](https://www.iplant.cn/info/Poaceae) | [Oplismenus](https://www.iplant.cn/info/Oplismenus) | 0.09 | 0.13 | - | - |
| 45 | [*Lonicera japonica*](https://www.iplant.cn/info/Lonicera%20japonica) | [Caprifoliaceae](https://www.iplant.cn/info/Caprifoliaceae) | [Lonicera](https://www.iplant.cn/info/Lonicera) | 0.02 | 0.02 | - | - |
| 46 | [*Cyclospermum leptophyllum*](https://www.iplant.cn/info/Cyclospermum%20leptophyllum) | [Apiaceae](https://www.iplant.cn/info/Apiaceae) | [Cyclospermum](https://www.iplant.cn/info/Cyclospermum) | 0.02 | - | - | - |
| 47 | [*Mazus pumilus*](https://www.iplant.cn/info/Mazus%20pumilus) | [Mazaceae](https://www.iplant.cn/info/Mazaceae) | [Mazus](https://www.iplant.cn/info/Mazus) | - | 0.04 | - | - |
| 48 | [*Vicia sepium*](https://www.iplant.cn/info/Vicia%20sepium) | [Fabaceae](https://www.iplant.cn/info/Fabaceae) | [Vicia](https://www.iplant.cn/info/Vicia) | - | 0.01 | - | - |
| 49 | [*Sambucus javanica*](https://www.iplant.cn/info/Sambucus%20javanica) | [Viburnaceae](https://www.iplant.cn/info/Viburnaceae) | [Sambucus](https://www.iplant.cn/info/Sambucus) | - | - | 0.05 | - |
| 50 | [*Cornus macrophylla*](https://www.iplant.cn/info/Cornus%20macrophylla) | [Cornaceae](https://www.iplant.cn/info/Cornaceae) | [Cornus](https://www.iplant.cn/info/Cornus) | - | - | 0.02 | - |
| 51 | [*Digitaria ciliaris*](https://www.iplant.cn/info/Digitaria%20ciliaris) | [Poaceae](https://www.iplant.cn/info/Poaceae) | [Digitaria](https://www.iplant.cn/info/Digitaria) | - | - | 0.02 | - |
| 52 | *B*[*idens pilosa*](https://www.iplant.cn/info/Bidens%20pilosa) | [Asteraceae](https://www.iplant.cn/info/Asteraceae) | [Bidens](https://www.iplant.cn/info/Bidens) | - | - | 0.02 | - |

Note: CK represents natural rainfall, MR represents moderate rainfall frequency enhanced treatment, HR represents heavy rainfall frequency enhanced treatment, and SR represents rainstorm frequency enhanced treatment.

Table S2 The selection criteria of the indicators to quantify EMF

| Single functions | Indicator | The functions of the indicator |
| --- | --- | --- |
| Plant productivity | BGM | The most important carbon sink in plants |
|  | AGM | The most important carbon sink in plants |
| Carbon cycle | SOC | Balance of soil organic carbon sinks and source processes |
|  | MBC | The most active component of soil organic carbon |
|  | S-SC | Reaction of soil carbon biological activity |
|  | Rs | Soil respiration is an important source of CO_2_ release into the atmosphere in terrestrial ecosystems |
|  | Rh | Soil microbial decomposition of litter and organic matter in soil |
|  | Ra | CO_2_ release from the plant roots and their associated rhizosphere |
| Nitrogen cycle | TN | Potential for continuous supply of nitrogen |
|  | NO_3_^-^-N | The available nitrogen components in the soil |
|  | IN | The retention capacity of the soil nitrogen |
|  | MBN | The most active component of soil nitrogen |
|  | NH_4_^+^-N | The available nitrogen components in the soil |
|  | AN | The available nitrogen components in the soil |
| Phosphorus cycle | TP | Potential for continuous supply of phosphorus |
|  | MBP | The most active component of soil phosphorus |
|  | ACP | Phosphorus-related enzymes that decompose organic phosphorus compounds |
|  | RP | The unavailable phosphorus components in the soil |
|  | PDEs | Phosphodiesterases affect the availability of soil |

Table S3 Abbreviation list

| Abbreviation | Academic words | Abbreviation | Academic words |
| --- | --- | --- | --- |
| CK | Natural rainfall treatment | CC | Carbon cycle |
| MR | Moderate rain frequency enhanced treatment | PP | Plant productivity |
| HR | Heavy rain frequency enhanced treatment | SOC | Soil organic carbon |
| SR | Rainstorm frequency enhanced treatment | MBC | Microbial biomass carbon |
| EMF | Ecosystem multifunctionality | S-SC | Soil sucrose |
| SEM | Structural equation model | MBN | Microbial biomass nitrogen |
| ABM | Aboveground biomass | NH_4_^+^-N | Ammonium nitrogen |
| BGM | Belowground biomass | AN | Alkali-hydrolyzable nitrogen |
| TN | Soil total nitrogen | TP | Soil total phosphorus |
| NO_3_^-^-N | Nitrate nitrogen | RP | Residual phosphorus |
| IN | Inorganic nitrogen | PDEs | Phosphodiesterases |
| NC | Nitrogen cycle | SWC | Soil water content |
| MBP | Microbial biomass phosphorus | pH | Potential of hydrogen |
| ACP | Acid phosphatase | PD | Plant phylogenetic diversity |
| PC | Phosphorus cycle | RF | Rainfall frequency |
| FD | Plant functional diversity | SD | Plant species diversity |
| SFD | Soil fungal diversity | SBD | Soil bacterial diversity |

**Reference**

Jenkinsone D.S., Vance D., Brookes P.C. (1987). An Extraction Method for Measuring Soil Microbial Biomass C. Soil Biology Biochemistry, 19(6): 703-707.

Lu, R. (1999). Analytical methods for soil and agricultural chemistry. China Agricultural Science and Technology Press: Beijing, 107-240.

Solaiman, Z. Measurement of microbial biomass and activity in soil, 2007.

Yue, P., Cui, X., Gong, Y., Li, K., Goulding, K., & Liu, X. (2018). Impact of elevated precipitation, nitrogen deposition and warming on soil respiration in a temperate desert. Biogeosciences, 15(7), 2007-2019.

Song, R., Han, X., Yang, Q., Zheng, Z. (2022). Effects of understory vegetation heterogeneity on soil organic carbon components in cunninghamia lanceolata plantation. Land, 11(12), 2300.

Oberson, A., Friesen, D.K., Morel C. Tiessen, H. (1997). Determination of phosphorus released by chloroform fumigation from microbial biomass in high P sorbing tropical soils. Soil Biology and Biochemistry, 29(9-10), 1579-1583.

Yang R., Xia X.M., Wang J.H. (2020). Dose and time-dependent response of single and combined artificial contamination of sulfamethazine and copper on soil enzymatic activities. Chemosphere, 250, 126161.

Lu W.X. (2004). Study on the method for the determination of nitric nitrogen, ammoniacal nitrogen and total nitrogen in plant. Spectiosopy and pedral Analysis, 24(2), 204-206.
